# Where Could Research on Immunosenescence Lead?

**DOI:** 10.3390/ijms20235906

**Published:** 2019-11-25

**Authors:** Mónica De la Fuente

**Affiliations:** Department of Genetics, Physiology and Microbiology (Animal Physiology Unit), School of Biology, Complutense University of Madrid (UCM), 28040 Madrid, Spain; mondelaf@bio.ucm.es

In the special issue of the *International Journal of Molecular Sciences* (ISSN 1422-0067) entitled: *Immunosenescence and related processes*, eight relevant articles are presented. As Guest Editor of this issue, it has been a pleasure to be involved with the publication of these stimulating proposals. Moreover, these manuscripts show aspects that are not very frequently considered in the study of immunosenescence, and they open new perspectives in this scientific field.

Although knowledge about the process of immunosenescence has increased in the last decades, many of the mechanisms involved in this process and associated pathologies remain unknown. Immunosenescence is characterized by age-related changes in molecular and cellular components of the immune system that ultimately lead to a general impairment of the immune response. Since an adequate function of immune cells is involved in the maintenance of health, and consequently in the rate of aging or biological age, immunosenescence is associated with an increased risk of morbidity and mortality [1]. Moreover, the values of several functions of these cells have been proposed as markers of health and the rate of aging of each individual, and for this reason, they could allow the prediction of his/her longevity [1,2,3].

This Special Issue is devoted to several age-related changes associated with immunosenescence, and takes into account the inflammatory and oxidative stress states of the organism in which the immune cells are involved [1].

In continuation, the most relevant contributions and suggestions for further research of each one of the eight manuscripts will be mentioned, following the order shown in Figure 1. For this, we are going to present three sections. The first contains those articles which provide a better understanding of the process of immunosenescence. The second proposes several possible markers of the aging process, and in the third section, an intervention using melatonin is discussed.

## 1. Helping to Understand the Aging Process and Immunosenescence

The articles by Konieczny and Arranz [4], by Garrido et al. [5], and by Min and Tatar [6] help in the better understanding of aging by focusing on several important aspects involving immunosenescence.

### 1.1. The Role of Stem Cells

The role of hematopoietic stem cells (HSCs) has been treated in an excellent way by Konieczny and Arranz [4]. HSC aging is characterized by reduced self-renewal, myeloid and platelet HSC skewing, and expanded clonal hematopoiesis. In this review, it is shown how these age-related HSC defects are reflected in insufficiencies in the progeny of differentiated cells, particularly in those of adaptive immunity, i.e., lymphoid progenitors. With aging, the myeloid skewing is performed at the expense of the lymphoid linage. A possible explanation could be that lymphocytes have a longer life span than myeloid cells, and infections and exposure to microbes will primarily influence myeloid-biased HSCs, with the objective of a rapid myeloid cell recovery. The potential underlying molecular mechanisms leading to HSC aging are also discussed in this article. This seems to be the result of increased oxidative stress, due to the accumulation of reactive oxygen species (ROS) and the consequent oxidative DNA damage, which is mainly caused by chronic exposure to infections, inflamm-aging, immunosenescence, and age-related changes in the HSC niche. Thus, HSC aging is multifactorial and we are only beginning to connect all the dots. Understanding the sequence of events and players leading to HSC aging will allow development of integrative and efficient strategies to improve hematopoiesis during aging.

### 1.2. A Confirmation of Oxi-Inflamm-Aging Associated with Immunosenescence and Lifespan

In agreement with the oxidation-inflammation theory of aging, the base of immunosenescence is the age-related chronic oxidative and inflammatory stress of immune cells. These stresses are involved in oxi-inflamm-aging of the organism, and consequently, in the rate of aging [1].

A confirmation of the oxidative-inflammatory basis of immunosenescence is given by the work carried out by Garrido and co-workers [5]. This study shows how immune cells from two models of adult mice with premature aging had lower values of antioxidant defenses and higher values of oxidants/pro-inflammatory cytokines than cells from the corresponding controls, and similar to those in cells from old animals. Thus, in both models of premature aging, one of them natural and the other with a genetic deletion, in which a premature immunosenescence and a shorter life span have been observed [1,7,8], the oxidative and inflammatory stress of peritoneal immune cells show similar values to those found in the corresponding chronologically old animals, analyzed in the same experiment. Moreover, oxidative stress was also detected in leukocytes from spleen and thymus. This fact corroborates that peritoneal immune cells are appropriate for studying the general redox state of the immune system. Given that a clear immunosenescence was detected in these peritoneal immune cells, these results confirm oxidative-inflammatory stress as the base of the deterioration of immune cell function. In addition, the present study corroborates the idea proposed in the theory of oxidation-inflammation that the immune system is involved in the rate of aging of the organism.

### 1.3. The Relevance of Innate Immunity in the Rate of Aging

The article by Min and Tatar [6] considers a very relevant subject. The authors used the *Drosophila* as a model to unravel the molecular mechanism of the process of immunosenescence. They review information about this process using this insect, focusing on the changes in insulin/IGF (insulin-like growth factor)-1 signaling hormones such a as juvenile hormone and 20-hydroxyecdysone. Moreover, a new perspective in the role of microbiota on the regulation of immunity and aging is discussed. This is a very novel aspect in the study of aging. In fact, the relevance of microbiota in the development and function of the homeostatic systems, such as the immune system, in mammals has currently been highlighted [9].

In addition, immunosenescence has frequently been focused on adaptive immunity. Although less recognition has been given to innate immunity, the important role of this in the overall decline in net immune function with aging has recently been accepted. Moreover, in the theory of oxidation-inflammation, which proposes that the immune cells are involved in the rate of aging [1], the relevance of innate cells, especially the phagocytes, in this process was suggested, and latterly confirmed by several experiments [10]. The fact that innate immunity exists in both vertebrates and invertebrates, unlike the cells of adaptive immunity, i.e., lymphocytes, which only appear in vertebrates, allows the universal application of this proposal [1].

*Drosophila* is an excellent model for studying the mechanisms of innate immunity, since, as the authors mentioned, “this fly possesses the basic recognition and signal transduction events of mammalian innate immunity without the added complication of adaptive immunity”.

In this section, other aspects that help to better understand the aging process and immunosenescence have also been considered. They are the influence of gender and infections on inflammatory state, as well as the relation of this with cognitive performance.

### 1.4. Are There Differences in Inflammation State Due to Gender and Cytomegalovirus (CMV) Infections in the Elderly Population? Relation between Inflammation and Cognitive Abilities

Following the idea of the presence of higher oxidation and inflammation in aging, two very related processes [11], it has been more easily accepted that, in humans, the advance of age is linked to persistent low-grade systemic inflammation, which is characterized by the increase in levels of pro-inflammatory cytokine circulation. This inflamm-aging may be different depending on the gender, as well as the number and kind of infections suffered by each individual. Virus infections, and especially the persistent infection with Cytomegalovirus (CMV), seem to be very related to the state of immunosenescence and, concretely, of inflammation [12].

The difference by sex in immunosenescence is an aspect very infrequently studied. If in adults, in general, females show a higher immune response than males with the advance of age, this difference is less marked and has been the subject of fewer studies [13]. Concretely, the influence of gender on the cytokine profile in elderly humans has rarely been investigated and the results obtained are contradictory. This aspect could give a better explanation of the different mean longevity of men and women. Moreover, it could be a key to the understanding of some mechanisms used by the organism to adapt the inflammatory state and to reach higher longevity.

The influence of gender on several inflammatory and anti-inflammatory mediators, focused on circulating cytokines, receptor antagonists, and soluble receptors, has been treated by Di Benedetto and co-workers [14].

Another relevant aspect is the bidirectional connection between the two homeostatic systems, i.e., the nervous and immune systems [15]. Thus, peripheral inflammation can affect brain function, and this fact is shown as changes in cognitive abilities such as memory (both working memory and episodic memory) and fluid intelligence. In fact, in the present article [14], different correlations between inflammatory and anti-inflammatory markers with cognitive performance have been detected depending on CMV-serostatus and gender. For this, both conditions should be considered in aging research and in interventional studies involving elderly people.

## 2. To Identify Biomarkers of Healthy and Pathological Aging

Currently, one relevant challenge in the studies of the aging process is to find markers that allow the detection of the rate of aging and the appearance of a pathology. In this context, the following three articles propose several different new markers.

### 2.1. Protein Carbamylation as a Possible Marker of the Rate of Aging

The article by Carracedo and co-workers [16] demonstrates that the amount of carbamylated proteins, which has been proposed as a hallmark of aging in tissues of mammals [17], is related to oxidative damage and the functional immunological signature, increasing in the peripheral blood of men in the 60–79 age group. In this experimental study, differences between men and women in the amount of protein carbamylation in plasma have been detected, for the first time. Thus, only men in the 60–79 age group presented higher carbamylated protein concentration, as well as oxidative damage to lipids (measured as malondialdehyde levels as a marker of oxidative stress [3]). Moreover, this study shows that the differences between the carbamylated proteins were not due to age-related changes in the total amount of proteins, a subject currently being discussed. Another relevant result of this work was that when men and women were classified by their immune function profiles, the carbamylated protein ratios were higher in those with an older functional immune signature (FIS), i.e., with a higher biological age. These results show that chronological age is not as important as biological age in the understanding of the value of markers of health and rate of aging. Although protein carbamylation could seem more relevant in detecting differences in the rate of aging, the results show its possible application as a marker of this, in spite of its limitations.

### 2.2. The Extracellular Vesicles

Picca and co-workers [18] proposed the use of extracellular vesicles (EVs) as a new target of study in several aspects of aging. Moreover, the EVs released by senescent cells, their content, traffic, and interaction with immune cells as a contribution to inflamm-aging is considered in this review. In addition, since mitochondria are key participants in the aging process, being the first target of age-related oxidative stress [1], it is very relevant to the better understanding of the role of these organelles in this process. The authors focus on the functional connection between lysosomes and mitochondria, not only to explain the generation of EVs, but also the relationship of the mitochondria–lysosomal axis and EV trafficking in the control of mitochondrial quality. On one hand, they confirm the role of EVs as novel biomarkers of the aging process. On the other, they suggest how the control of the materials transported into these EVs, as well as their traffic as delivery systems for therapeutics against age-related conditions, allows extended health and lifespan. Thus, although the mechanisms responsible for the coordination between EV traffic and the mitochondrial–lysosomal axis are still unclear, it is evident that their disruption is implicated in the aging process, and the knowledge of these mechanisms may allow the development of innovative and personalized anti-aging interventions.

### 2.3. Lymphoproliferative Function and Oxidative Stress Parameters as Markers of Parkinson’s Disease

In pathological states associated with oxidation and inflammation (depression and metabolic, cardiovascular, or neurodegenerative diseases, among others), the process of immunosenescence and the mechanisms involved in the spread of immune cell alterations to the other cells of the body have hardly been studied. Parkinson’s disease (PD) is the second most prevalent age-related neurodegenerative disorder. Currently, considerable efforts are being carried out to detect peripheral biomarkers that allow the diagnosis of neurodegenerative diseases such Alzheimer’s disease [19] and PD, especially in early stages of clinical development. This would allow greater effectivity of interventions in these neuropathologies, which today have no effective treatments. Vida and co-workers [20] analyzed several immune functions of neutrophils and lymphocytes of peripheral blood, as well as oxidative stress and damage parameters in whole blood cells of PD stage 2 patients. They observed an accelerated immunosenescence in these patients and proposed the proliferative response to mitogens of lymphocytes as a good marker of the immune alterations at this stage of the pathology. Moreover, several parameters of oxidative stress were increased in the blood cells of these patients. This is the first approach to suggest possible biomarkers during the early stages of PD. To extend and validate these markers, as well as to corroborate if they are also useful in other stage of this disease, are future challenges.

## 3. A Possible Intervention to Control Inflammatory Stress

The kind and number of possible interventions that may improve the aging process are numerous. A frequently studied molecule in this context is melatonin. This hormone, with many positive effects, has been widely used in the control of aging due to its antioxidant and anti-inflammatory properties, together with its cellular protective capacity [21].

### Melatonin on Inflammation in Brain Ischemia

Strokes are one of the leading causes of death in developed countries. In this pathological process, the ischemic injury, associated with oxidative and inflammatory stress, in which the immune cells are involved, plays a relevant role in the death of brain cells. The therapeutic options for this acute ischemia are very limited and to find successful treatments, especially in elderly patients, is one of the major challenges in medicine. Melatonin, as a relevant antioxidant, has been proposed as a neuroprotective agent, especially in brain ischemia [22]. Rancan and co-workers [23] showed the protective role of melatonin on the inflammatory and apoptotic response of an ischemic injury in the brain of old rats. Although the effects are similar using previous or post-surgery treatment with this antioxidant, they showed to a lesser extent in the first case. This study opens with a discussion about when the best time would be to use an antioxidant in a treatment. In fact, this represents one of the reasons for the controversial results obtained using antioxidants, especially in aging. The results depend on several factors, such as the amount, the extent, and the moment of administration, among others. This is another scientific field in which much further study is desirable.

## 4. Conclusions

All eight articles cover new aspects related to immunosenescence and suggest other future relevant studies in this field.

In all of these studies, in a direct or sometimes indirect way, the basis of oxidative and inflammatory stresses underlying immunosenescence and the aging process have been considered. This highlights the relevance of these mechanisms as the cause of aging, in contrast to certain criticism that this idea has received in recent years [24].

Currently, it is accepted that immune cells are markers of health and can be involved not only in the maintenance of health, but also in the rate of aging and, consequently, in the life span of each individual. Thus, it is reasonable to think that everything that improves the knowledge of these cells and their related processes, especially in aging, could be very relevant to achieve a healthy longevity.

## Figures and Tables

**Figure 1:**
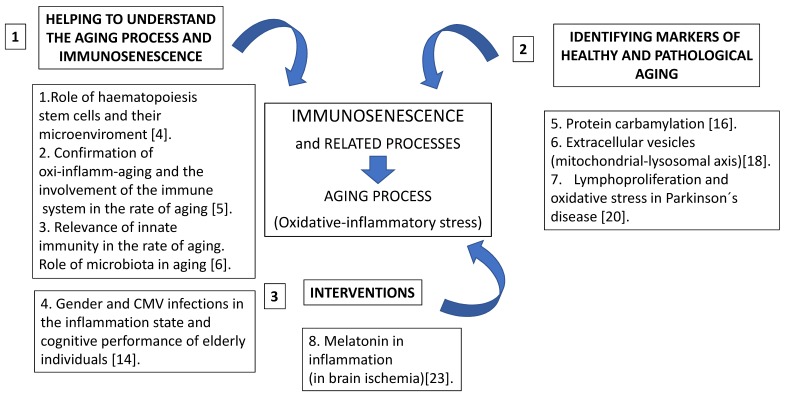
Contributions of each work to the field of Immunosenescence and related processes.
